# Dual-mode electrochemiluminescence and electrochemical sensor for alpha-fetoprotein detection in human serum based on vertically ordered mesoporous silica films

**DOI:** 10.3389/fchem.2022.1023998

**Published:** 2022-11-07

**Authors:** Haiyun Chen, Jie Huang, Rongjing Zhang, Fei Yan

**Affiliations:** ^1^ Shanxi Bethune Hospital, Shanxi Academy of Medical Sciences, Tongji Shanxi Hospital, Third Hospital of Shanxi Medical University, Taiyuan, China; ^2^ Tongji Hospital, Tongji Medical College, Huazhong University of Science and Technology, Wuhan, China; ^3^ Department of Chemistry, Key Laboratory of Surface and Interface Science of Polymer Materials of Zhejiang Province, Zhejiang Sci-Tech University, Hangzhou, China

**Keywords:** vertically ordered mesoporous silica film, alpha-fetoprotein, dual-mode detection, electrochemiluminescence, electrochemistry, immunosensor

## Abstract

In this study, we demonstrated the highly sensitive detection of alpha-fetoprotein (AFP) by electrochemiluminescence (ECL) and electrochemistry (EC) based on the gated transport of the bifunctional probe (tris(1,10-phenanthroline) ruthenium (II) chloride, Ru (phen)_3_Cl_2_) into the nanochannels of vertically ordered mesoporous silica films (VMSFs). Due to the negatively charged surface and ultrasmall pore size, VMSF displays a signal amplification effect on Ru (phen)_3_Cl_2_ and is suitable for the construction of sensors with excellent sensitivity. With the linkage of (3-glycidyloxypropyl) trimethoxysilane, the anti-AFP antibody could covalently bind to the external surface of VMSF, generating a highly specific recognized sensing interface toward AFP. When AFP is presented, the formed immunocomplex hinders the diffusion of Ru (phen)_3_Cl_2_ to the underlying electrode surface, resulting in a decreased ECL or EC response. The dual-mode detection of AFP is achieved with a relatively low limit of detection (0.56 fg/ml for ECL and 4.5 pg/ml for EC) and a wide linear range (10 fg/ml∼1 μg/ml for ECL and 10 pg/ml∼1 μg/ml for EC). Moreover, owing to the inherent anti-fouling property of VMSF, satisfactory results in the analysis of human serum were obtained, showing the great potential of the designed strategy in clinical diagnosis.

## 1 Introduction

Cancer has become the first or second leading cause of death before the age of 70 in 61% of countries, according to an investigation from the World Health Organization (Sung et al., 2021). Hepatocellular carcinoma (HCC) is one of the most common malignant tumors with high malignancy and poor prognosis (Suriapranata et al., 2010). Alpha-fetoprotein (AFP), synthesized by fetal hepatocytes and yolk sac with a molecular weight of approximately 70 kDa, has been considered a critical biomarker for HCC (Zhang et al., 2016). Generally, the AFP content in the serum of newborns ranges from 10 to 50 μg/ml and decreases gradually after birth. As the AFP could not be produced in mature and healthy hepatocytes, the AFP level in the serum of a healthy adult is below 20 ng/ml (Dutta et al., 2021). Abnormal AFP levels (usually higher than 500 ng/ml) are highly relative to HCC, metastatic cancers of the liver, or many other cancers (e.g., germ cell tumors, pancreatic tumors, and carcinoma of the gallbladder) (Dai et al., 2012; Kal-Koshvandi, 2020). Therefore, it is crucial to develop accurate and sensitive detection methods for AFP to facilitate the early diagnosis and the evaluation of treatment effectiveness.

To date, numerous methods have been reported for the detection of AFP and have achieved satisfactory results, such as enzyme-linked immunosorbent assay (Liu et al., 2021), surface plasmon resonance (Teramura and Iwata, 2007), fluorescence immunoassay (Ma R. et al., 2019), colorimetry (Ma F. et al., 2019; Li et al., 2022), surface-enhanced Raman scattering (Ma et al., 2017), and chemiluminescence immunoassay (Yang et al., 2008). However, these techniques often require complex operations, expensive instruments, professional technicians, and long detection time. With the advantages of high sensitivity, rapid responses, simple operations, and easy portability, electrochemiluminescence (ECL) (Wang et al., 2018; Zheng et al., 2021) and electrochemistry (EC) (Liao et al., 2021; Gong et al., 2022a; Zhou et al., 2022a; Yan et al., 2022; Zhang et al., 2022) have been successfully applied to construct biosensors. The combination of ECL and EC to construct ECL/EC dual-mode sensors is able to greatly improve the reliability and accuracy and has aroused considerable attention (Gong et al., 2022b). To the best of our knowledge, the utilization of ECL/EC dual-mode sensors for AFP detection has not been reported yet.

Porous materials have played important roles in the construction of the sensing interface (Liu et al., 2018; Cui et al., 2020; Zhang et al., 2021; Cui et al., 2021; Liu et al., 2022; Su et al., 2022). Vertically ordered mesoporous silica films (VMSFs) consisting of perpendicular nanochannels, uniform and ultrasmall pore size, negative surface charge, and high porosity show high molecular permeability, charge permselectivity, and anti-fouling capacity, which have been widely used as a modified layer for the prominent improvement of electrode performance (Zhou et al., 2019; Walcarius, 2021; Zhou et al., 2022b; Zheng et al., 2022; Zhu et al., 2022). VMSF has been proven to preconcentrate the cationic ECL probe [(tris(2,2′-bipyridyl)] ruthenium [Ru (bpy)_3_^2+^)] in the aqueous solution through the electrostatic effect, greatly reducing the amount of Ru (bpy)_3_^2+^ and simultaneously enhancing the ECL intensity by two orders of magnitude (Zhou et al., 2015; Luo et al., 2022; Wei et al., 2022). Moreover, by tailoring recognitive molecules on the inner channel walls or outer surface, VMSF-based ECL or EC sensors have been designed to detect a variety of targets, such as ions (Cheng et al., 2018; Yan et al., 2020), small biological molecules (Ma et al., 2022a; Zhou et al., 2022c; Wang et al., 2022), DNA (Saadaoui et al., 2016), antibody (Gong et al., 2022c), antigen (Ma et al., 2022b), and cancer cell (Wu et al., 2015). Therefore, VMSF is very suitable for the construction of ECL/EC dual-mode sensors and shows great potential in the direct analysis of complex real samples without tedious sample pretreatments.

In this work, highly sensitive and low-cost detection of AFP was realized by both ECL and EC modes based on the amplified effect of VMSF. VMSF grown onto the indium tin oxide (ITO) electrode with negative surface charge was able to electrostatically preconcentrate the cationic bifunctional probe (tris(1,10-phenanthroline) ruthenium (II) chloride, Ru (phen)_3_Cl_2_), leading to the remarkably enhanced ECL intensity and simultaneously decreasing the amount of Ru (phen)_3_Cl_2_. The anti-AFP antibody was covalently modified onto the external surface of VMSF, and this was performed by using a (3-glycidyloxypropyl) trimethoxysilane linker, exhibiting good specificity toward AFP recognition. Because the formed immunocomplex hinders the diffusion of Ru (phen)_3_Cl_2_ to the underlying electrode surface, decreased ECL or EC response was recorded, and the quantitative determination of AFP was achieved. Furthermore, the detection of AFP in human serum has also been studied using the proposed dual-mode sensor.

## 2 Materials and methods

### 2.1 Chemicals and materials

AFP, anti-AFP antibody, carcinoembryonic antigen (CEA), and carcinoma antigen 199 (CA199) were purchased from Beijing KEY-BIO Biotech Co., Ltd. (China). Serum amyloid A (SAA) protein was bought from Nanjing Okay Biotechnology Co., Ltd. (China). S100 calcium-binding protein *β* (S100*β*) was purchased from Proteintech (China). Potassium ferricyanide (K_3_ [Fe(CN)_6_]), potassium ferrocyanide (K_4_ [Fe(CN)_6_]), potassium hydrogen phthalate (KHP), bovine serum albumin (BSA), potassium chloride (KCl), tetraethoxysilane (TEOS), cetyltrimethylammonium bromide (CTAB), sodium hydroxide (NaOH), sodium phosphate monobasic dihydrate (NaH_2_PO_4_·2H_2_O), sodium phosphate dibasic dodecahydrate (Na_2_HPO_4_·12H_2_O), hexaammineruthenium (III) chloride (Ru(NH_3_)_6_Cl_3_), (3-glycidyloxypropyl) trimethoxysilane (GPTMS), fetal calf serum, and tripropylamine (TPA) were obtained from Aladdin Biochemical Technology Co., Ltd. (China). Ethanol (99.8%), acetone, sodium nitrate (NaNO_3_), and hydrochloric acid were purchased from Hangzhou Gaojing Fine Chemical Co., Ltd. (China). Tris (1,10-phenanthroline) ruthenium (II) chloride dihydrate (Ru (phen)_3_Cl_2_·2H_2_O) was purchased from Shanghai Yien Chemical Technology Co., Ltd. (China). All the chemicals were of analytical grade and used without further purification. Ultrapure water (18.2 MΩ cm) was used to prepare all aqueous solutions throughout the work. ITO glasses (< 17 Ω/square, thickness: 100 ± 20 nm) were purchased from Zhuhai Kaivo Optoelectronic Technology, and washed with 1 M NaOH aqueous solution overnight followed by subsequent treatment with acetone, ethanol, and deionized water under ultrasonic for 30 min.

### 2.2 Measurements and instrumentations

The morphology of VMSF/ITO was investigated using a transmission electron microscope (TEM, JEM-2100, JEOL, Japan) and scanning electron microscope (SEM, SU8010, Hitachi, Japan) with an acceleration voltage of 200 kV and 10 kV, respectively. All the electrochemical procedures containing cyclic voltammetry (CV), differential pulse voltammetry (DPV), and electrochemical impedance spectroscopy (EIS) were performed on an Autolab electrochemical workstation (Metrohm, PGSTAT302N, Switzerland). Electrochemiluminescence (ECL) measurements were conducted on an MPI-E II ECL analytical system (Xi’an Remax Electronic Science and Technology Co., Ltd.). The voltage of the photomultiplier tube (PMT) was set at 500 V. A conventional three-electrode system was applied for both ECL and EC measurements, where a bare or modified ITO electrode was used as the working electrode, a platinum wire was used as the counter electrode, and an Ag/AgCl electrode (saturated with KCl) was used as the reference electrode.

### 2.3 Preparation of the VMSF/ITO electrode

Modification of VMSF on the ITO electrode surface is realized by using an electrochemically assisted self-assembly (EASA) method as previously reported (Walcarius et al., 2007). Briefly, a precursor solution was obtained by mixing 2.833 g of TEOS and 1.585 g of CTAB with 20 ml ethanol and 20 ml 0.1 M NaNO_3_ with pH adjusted by HCl to 2.6 and further stirring for 2.5 h to pre-hydrolyze. A clean ITO electrode was immersed into the precursor solution as a working electrode, and a constant cathodic current density (–0.70 mA cm^−2^) was applied to the ITO with Ag/AgCl (saturated KCl) as the reference electrode and a platinum plate (2 cm × 4 cm) as the counter electrode. Then, the as-prepared electrode was rapidly taken out and washed with a great deal of ultrapure water, and dried in a nitrogen stream. After further aging at 120°C overnight, the obtained electrode was denoted as SM@VMSF/ITO, since there existed CTAB surfactant micelles (SMs) inside the nanochannels. SM can be removed by simply stirring the SM@VMSF/ITO electrode in 0.1 M HCl–ethanol solution for 5 min. To discriminate, this electrode was named VMSF/ITO. Prior to use, VMSF/ITO electrodes were treated with Scotch tape to remove aggregates on the surface of VMSF (Moehl et al., 2022).

### 2.4 Preparation of an ECL/EC dual-mode immunosensor

**SCHEME 1:**
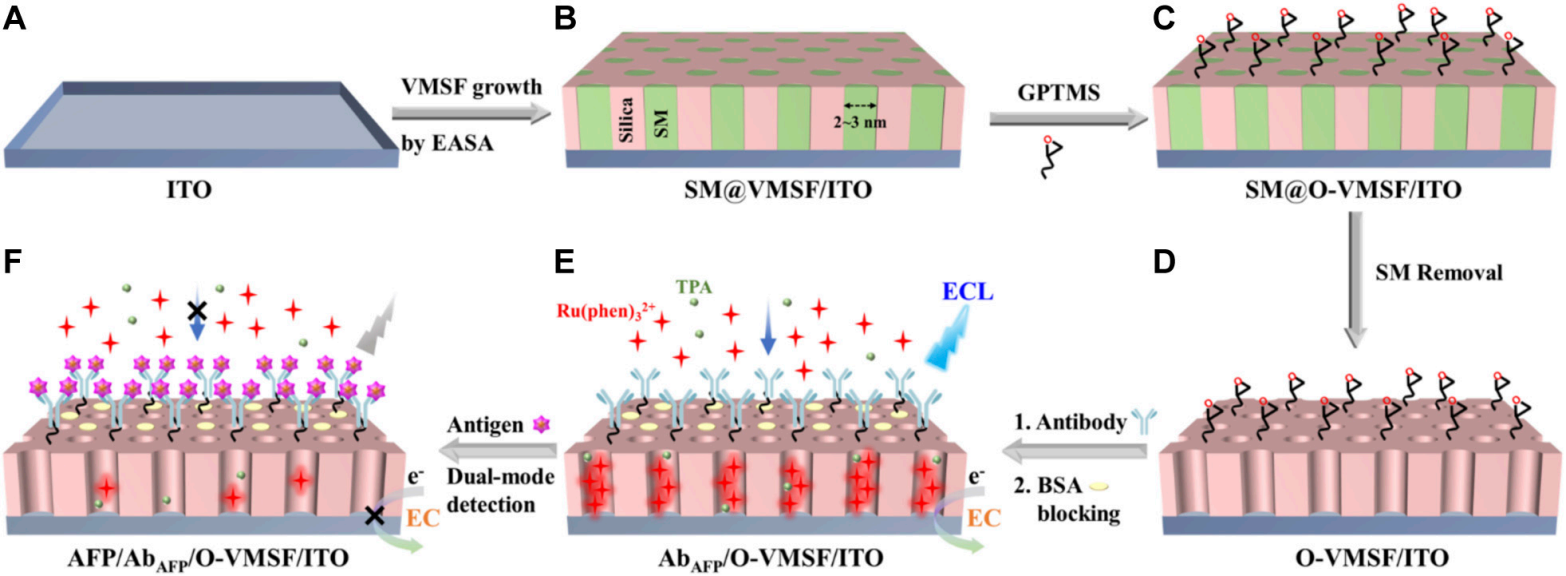
Illustration of the fabrication of the immunosensing interface based on the VMSF/ITO electrode **(A-E)** and the following label-free ECL and EC detection of AFP **(E,F)**.

As shown in Scheme 1, the VMSF/ITO electrode is used as the supporting substrate for the construction of the immunosensors. VMSF possesses abundant silanol groups and can offer easy modification with silane-coupling reagents containing reactive groups. GPTMS was covalently linked on the outer surface and the entry of nanochannels by treating SM@VMSF/ITO with 2.26 mM GPTMS–ethanol solution for 1 h. Then, the resulting electrode was rinsed thoroughly with ultrapure water. By immersing in 0.1 M HCl–ethanol solution and stirring for 5 min, epoxy-functionalized VMSF/ITO with open channels was obtained, termed O-VMSF/ITO. The immunorecognitive interface was fabricated by drop-coating anti-AFP antibody (Ab_AFP_, 50 μl and 10 μg/ml) on the O-VMSF/ITO electrode surface and incubation at 37°C for 90 min through the nucleophilic reaction between aminos of the antibody and epoxy groups on the electrode surface, followed by thorough rinsing with PBS (0.01 M, pH 7.4) to remove the unbound antibodies. After blocking the non-specific sites with BSA (1%, wt%) for 60 min, the as-prepared immunosensor was denoted as Ab_AFP_/O-VMSF/ITO.

### 2.5 Detection of AFP

Determination of AFP was achieved by ECL and EC methods. Typically, the Ab_AFP_/O-VMSF/ITO sensor was incubated with different concentrations of AFP at 37°C for 60 min. After being thoroughly washed with PBS (0.01 M, pH = 7.4), AFP/Ab_AFP_/O-VMSF/ITO with different amounts of the immune complex on the electrode surface was obtained. The ECL intensities or the anodic peak currents of the immunosensor before and after AFP binding were measured. For ECL detection, Ab_AFP_/O-VMSF/ITO or AFP/Ab_AFP_/O-VMSF/ITO was immersed in 0.01 M PBS (pH = 7.4) containing 10 μM Ru (phen)_3_^2+^ and 3 mM TPA for 10 min to enrich the luminophores. It is important to note that 3 mM TPA used here is excess in order to ensure continuous and stable ECL emission (Ma et al., 2022b). Then, ECL signals were recorded by continuous cyclic scanning between 0 and 1.25 V with a scan rate of 100 mV/s. For EC detection, Ab_AFP_/O-VMSF/ITO or AFP/Ab_AFP_/O-VMSF/ITO was immersed in 0.01 M PBS containing 0.5 mM Ru (phen)_3_^2+^ for 5 min to enrich the electrochemical probe. Then, current responses were recorded using the DPV technique. For real sample analysis, human serum was diluted 50 times with PBS (0.01 M, pH 7.4) and directly determined using the developed immunosensor.

## 3 Results and discussion

### 3.1 Characterization of the VMSF/ITO electrode

**FIGURE 1:**
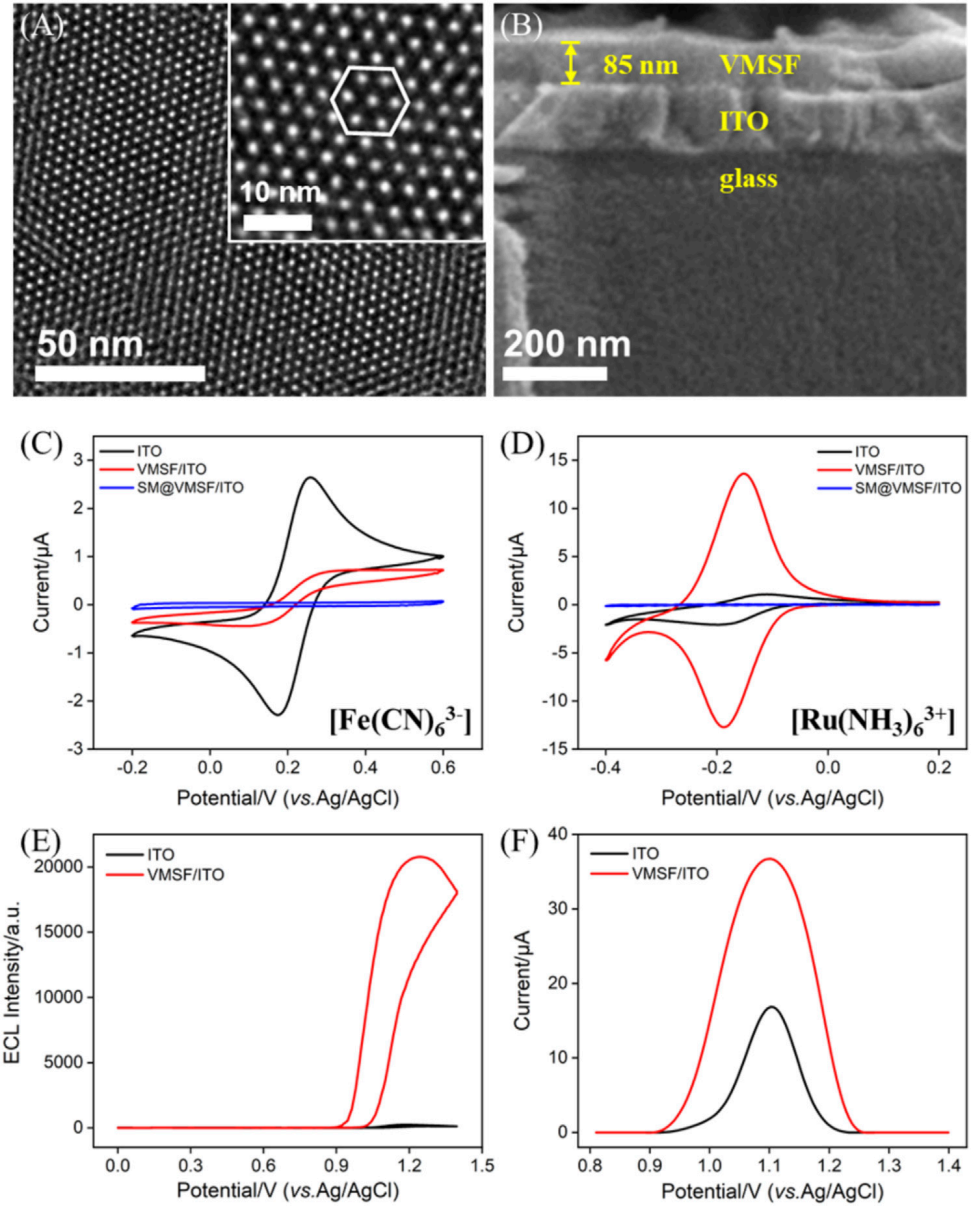
Top-view TEM **(A)** and section-view SEM **(B)** images of VMSF. The inset in **(A)** corresponds to the magnified image. CV curves of ITO, VMSF/ITO, and SM@VMSF/ITO obtained in **(C)** 0.05 M KHP containing 50 μM Fe(CN)_6_^3–^ (adjusting pH to 7.4) and **(D)** 0.05 M PBS (pH=7.4) containing 50 μM Ru(NH_3_)_6_^3+^. ECL **(E)** and DPV **(F)** curves of bare ITO and VMSF/ITO electrodes obtained in 0.01 M PBS (pH = 7.4). The detected solution in **(E)** contains 10 μM Ru (phen)_3_^2+^ and 3 mM TPA and that in **(F)** contains 0.5 mM Ru (phen)_3_^2+^.

Growth of VMSF on the ITO substrate was obtained by the EASA method, as shown in Scheme 1. EASA is a simple and rapid method used for the growth of VMSF with good reproducibility on conductive substrates. In the EASA method, the condensation of the siloxane precursor (TEOS) is triggered around the CTAB surfactant micelle (SM) template by applying a cathode potential to the electrodes to generate a hydroxide ion (OH^−^) catalyst through the reduction of water and nitrate ions (NO_3_^−^) (Ma et al., 2022b). The as-prepared SM@VMSF/ITO contains SM inside the channels, which can be removed by soaking it in HCl–ethanol to get the channels open (Giordano et al., 2017). The morphology of VMSF/ITO was first investigated by TEM and SEM. As shown in the TEM image (Figure 1A), VMSF has numerous hexagonally packed nanopores with a uniform diameter of 2–3 nm and exhibits a high porosity of ∼45%. The cross-sectional SEM image of VMSF/ITO shows that the thickness of VMSF is homogeneous and about 85 nm (Figure 1B). Two kinds of electrochemical probes (Fe(CN)_6_^3–^ and Ru(NH_3_)_6_^3+^) with different charges were selected to prove the integrity and permeability of VMSF. Figures 1C,D depict the electrochemical behaviors of probes on the three types of electrodes. Owing to the fact that CTAB SM inside the channels hinders the transfer of these two probes from the bulk solution to the electrode surface, no faradic currents but only capacitive currents were observed at the SM@VMSF/ITO electrode, indicating the integrity of VMSF without cracks or defects. After the removal of SM, at the pH of 7.4, the redox peak currents of Fe(CN)_6_^3–^ were significantly suppressed at the VMSF/ITO electrode, while those of Ru(NH_3_)_6_^3+^ remarkably enhanced compared with bare ITO. This prominent charge-based permeability ascribes to the deprotonation of abundant silanols (p*K*_a_∼2) inside the nanochannels, suggesting that the as-prepared VMSF/ITO facilitates the electrostatic enrichment of positively charged species. Then, we investigated the enhancement effect of ECL/EC signals of the bifunctional probe Ru (phen)_3_^2+^ with the help of VMSF, and the results are shown in Figures 1E,F. As seen, the ECL intensity of 10 μM Ru (phen)_3_^2+^ obtained at the VMSF/ITO electrode is two orders of magnitude higher than that of the bare ITO electrode (Figure 1E), and the redox peak currents of 0.5 mM Ru (phen)_3_^2+^ obtained at the VMSF/ITO electrode is two-fold higher than that of the bare ITO electrode (Figure 1F **)**. The aforementioned results suggest that VMSF/ITO with charge selectivity has great potential for the construction of Ru (phen)_3_^2+^-based electrochemical/electrochemiluminescent sensors with high sensitivity.

### 3.2 Characterization of the ECL/EC dual-mode immunosensor

**FIGURE 2:**
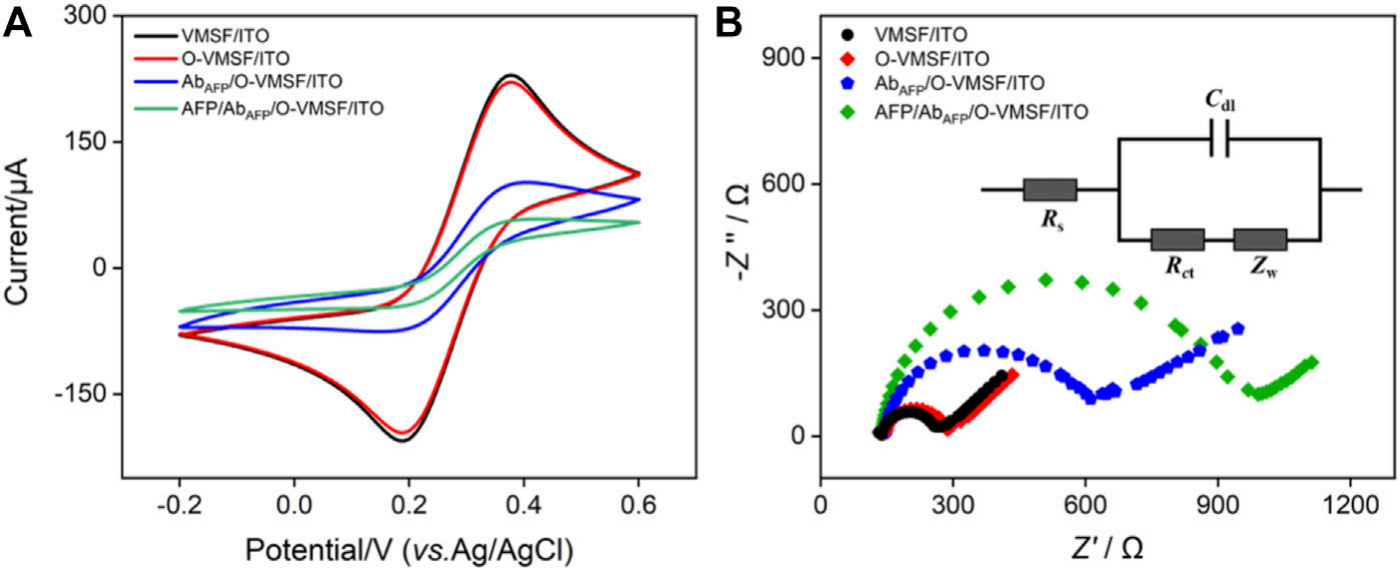
CV curves **(A)** and EIS plots **(B)** of VMSF/ITO, O-VMSF/ITO, Ab_AFP_/O-VMSF/ITO, and AFP/Ab_AFP_/O-VMSF/ITO electrodes obtained in 0.1 M KCl containing 2.5 mM Fe(CN)_6_^3–/4–^. The inset in **(B)** is the equivalent circuit. The concentration of AFP is 1 ng/ml.

The modification procedures and feasibility of the construction of the immunosensor were proved by electrochemical methods including CV and EIS. Figure 2 shows the CV curves and EIS plots obtained on various electrodes in 2.5 mM Fe(CN)_6_^3–/4–^ solution. As seen, the redox peak currents of the O-VMSF/ITO electrode are similar to those of VMSF/ITO with only a slight decrease, which is because the GPTMS monolayer being covalently linked on the outer surface and the entry of silica nanochannels instead of the inner nanochannels and also suggests that the linkage of the epoxy group on the outer surface of VMSF hardly affects the mass transfer (Figure 2A). After the immobilization of Ab_AFP_, the redox peak currents underwent a significant decline, and the peak-to-peak difference became larger. Supplementary Figure S1 compares the CV curves of VMSF/ITO before and after incubation with Ab_AFP_. No obvious change is observed, indirectly indicating the presence of GPTMS entities on the outer surface of O-VMSF/ITO and their important role in Ab_AFP_ immobilization. When AFP is present, AFP can bind specifically to Ab_AFP_ to form the immunocomplex on the electrode surface, leading to further decreased redox peak currents and increased peak-to-peak difference. This is because the Ab_AFP_ or the immunocomplex with non-conductive and large-sized characteristics could hamper the electron transfer of the electrochemical probes on the electrode interface. EIS is also applied to investigate the interface properties during the sensor construction. As depicted in Figure 2B, there are two segments in each EIS plot: a semicircle in the high-frequency region representing electron transfer-limited processes and a linear part in the low-frequency region representing diffusion-limited processes (Lu et al., 2018). The inset demonstrates the equivalent circuit, which consists of the solution resistance (*R*_s_), double-layer capacitance (*C*_dl_), Warburg impedance (*Z*_w_), and apparent charge-transfer resistance (*R*_ct_). The equivalent diameter of the semicircle in the high-frequency region is equal to the apparent charge-transfer resistance *R*_ct_, which increased from 128 Ω for ITO to 140 Ω for O-VMSF/ITO, 499 Ω for Ab_AFP_/O-VMSF/ITO, and 2945 Ω for AFP/Ab_AFP_/O-VMSF/ITO. All these results confirm the successful construction of the immunosensor.

### 3.3 ECL detection of AFP

**FIGURE 3:**
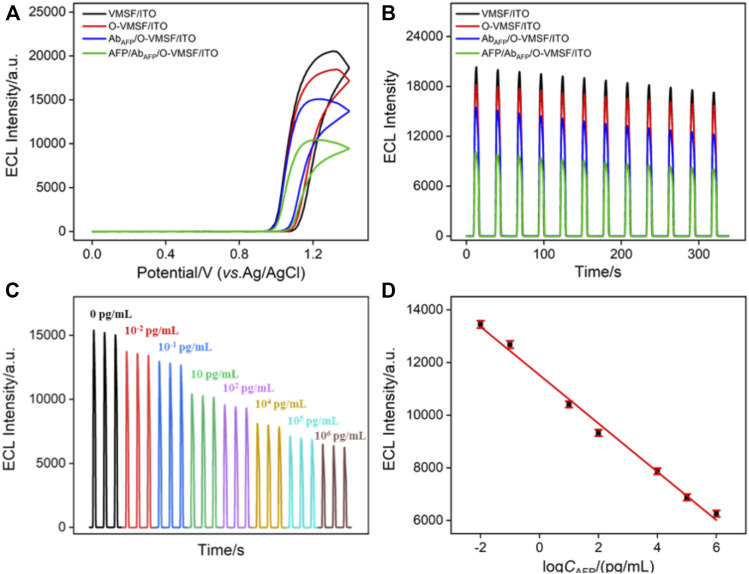
ECL–potential **(A)** and ECL–time **(B)** curves obtained at different electrodes. The concentration of AFP is 1 pg/ml. **(C)** ECL responses of the ECL immunosensor in the presence of different concentrations of AFP. **(D)** Corresponding calibration curve. Error bars represent the standard deviation of three measurements. The electrolyte is 0.01 M PBS (pH = 7.4) containing 10 μM Ru (phen)_3_^2+^ and 3 mM TPA. The PMT voltage is 500 V.

Figures 3A,B compare the ECL–potential, and ECL–time curves of the Ru (phen)_3_^2+^/TPA co-reactant system at various electrodes, namely, VMSF/ITO, O-VMSF/ITO, Ab_AFP_/O-VMSF/ITO, and AFP/Ab_AFP_/O-VMSF/ITO electrodes. Similar to the aforementioned CV and EIS results, decreased ECL intensity is observed for each fabricated step and further incubation with 1 pg/ml AFP. Also, the resulting ECL sensor (Ab_AFP_/O-VMSF/ITO) has satisfactory stability under continuous scanning (relative standard deviation (RSD), less than 7%) before and after the detection of AFP. These results indicate that the proposed ECL immunosensor has the ability to detect AFP. Based on the AFP-controlled transport of Ru (phen)_3_^2+^ into the silica nanochannels, the as-prepared Ab_AFP_/O-VMSF/ITO immunosensor was applied to detect various concentrations of AFP in the buffer solution, and the results are shown in Figures 3C,D. As seen, the obtained ECL signals gradually decrease with the increasing AFP concentration, and the corresponding calibration curve shows a good linear relationship between ECL intensity (*I*_ECL_) and the logarithmic value of AFP concentration (log*C*_AFP_) ranging from 10 fg/ml to 1 μg/ml (*I*_ECL_ = −919 log*C*_AFP_ + 11,524, *R* ^2^ = 0.992). The limit of detection (LOD) is calculated to be 0.56 fg/ml (S/N = 3). Moreover, Table 1 summarizes the detection performance of various ECL methods for the detection of AFP. Although excellent analytical performance is achieved by these listed sensors, most of them are based on the label strategy and require laborious procedures. Without the need for synthesizing complex nanomaterials or tedious labeling, the proposed immunosensor exhibits a wider linear range and a much lower LOD.

**TABLE 1:** Comparison of the analytical performances of various electroanalytical methods for the determination of AFP.

Electrode	Method	Classification	Linear range (pg/ml)	LOD (pg/ml)	Reference
Ab_2_-ZnO-Ru and Ab_1_-AuNPs-rGO/GCE	ECL	Labeled	40–5×10^5^	31	Zhao et al. (2018)
Ru-Si@AuNPs/PLL/rGO/Ab_2_ and Ab_1_/AuNPs/GCE	ECL	Labeled	3–5×10^5^	0.5	Liu et al. (2014)
PAADs@CNDs@Ab_2_ and Ab_1_/C_60_/rGO/GCE	ECL	Labeled	10^−3^–8×10^4^	3.3×10^−4^	Zhang et al. (2016)
HRP/AuNRDs/Ab_2_ and CdS:Eu QDs/AuNPs/rGO/GCE	ECL	Labeled	0.05–10^4^	0.05	Feng et al. (2015)
CdSe/Ab_2_ and Ab_1_/ABA/GCE	ECL	Labeled	0.05–10^2^	0.01	Cui et al. (2016)
CNTs@PNFs/CS/GCE	ECL	Label-free	0.1–1.6×10^5^	0.09	Zou et al. (2017)
CNDs-Nafion/GCE	ECL	Label-free	0.1–3.2×10^5^	0.1	Dai et al. (2012)
AuNPs/*g*-C_3_N_4_/GCE	ECL	Label-free	1–5×10^3^	0.5	Dai et al. (2014)
PPy-MO DMIP/FTO	EIS	Label-free	10–10^4^	3.3	Zheng et al. (2016)
AF/PDA/Ab_2_ and Ab_1_/GE	CA	Labeled	0.5–10^3^	0.01	Taheri et al. (2022)
Fc/GO-DETA/Ab_2_ and Ab_1_/AuNPs-rGO/GCE	DPV	Labeled	0.35–3.5×10^4^	0.014	Gong et al. (2021)
Au@CeO_2_ YSNs-Ab_2_ and Ab_1_/AuNPs/GCE	LSV	Labeled	0.1–2×10^5^	0.035	Liao et al. (2021)
Ab/N-GQD/SWCNHs/GCE	CV	Label-free	1–2×10^5^	0.25	Dutta et al. (2021)
Ab/p-PANI/GCE	DPV	Label-free	0.01–10^3^	3.7×10^−3^	Wang et al. (2021)
Ab/O-VMSF/ITO	ECL	Label-free	10^−2^–10^6^	5.6×10^−4^	This work
	EC		10–10^6^	4.5	

Ab, antibody; AuNPs, gold nanoparticles; rGO, reduced graphene oxide; GCE, glassy carbon electrode; PLL, poly- *l* -lysine; PAADs, poly (amidoamine) dendrimers; CNDs, carbon nanodots; AuNRDs, gold nanorods; QDs, quantum dots; ABA, *p*-aminobenzoic acid; CNTs, carbon nanotubes; PNFs, produced nanofibers; CS, chitosan; *g*-C_3_N_4_, graphite-like carbon nitride; PPy, polypyrrole; MO, methyl orange; DMIP, dual-template molecularly imprinted polymer; FTO, F-doped tin oxide; EIS, electrochemical impedance spectroscopy; AF, aminoferrocene; PDA, polydopamine; GE, gold disk electrode; CA, chronoamperometry; YSNs, yolk shell nanostructures; AuNPs, gold nanoparticles; GCE, glassy carbon electrode; LSV, linear sweep voltammetry; N-GQD, nitrogen-doped graphene quantum dot; SWCNHs, single-walled carbon nanohorns; CV, cyclic voltammetry; p-PANI, porous polyaniline.

### 3.4 EC detection of AFP

**FIGURE 4:**
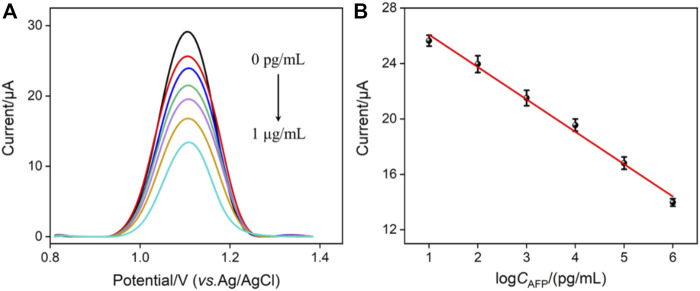
**(A)** DPV curves of the immunosensor in the presence of different concentrations of AFP. **(B)** Corresponding calibration curve. Error bars represent the standard deviation of three measurements. The electrolyte is 0.01 M PBS (pH = 7.4) containing 0.5 mM Ru (phen)_3_^2+^.

Arising from the inherent redox characteristic of Ru (phen)_3_^2+^, the proposed immunosensor was also used to quantitatively detect AFP by the EC mode. Figure 4A shows the DPV responses of the Ab_AFP_/O-VMSF/ITO electrode to the different concentrations of AFP. Upon increasing the concentration of AFP, the decreased anodic peak currents were obviously displayed, showing a good linear relationship with the log*C*_AFP_ ranging from 10 pg/ml to 1 μg/ml (*I*_EC_ = −2.33 log*C*_AFP_ + 28.41, *R* ^2^ = 0.991). The LOD is calculated to be 4.5 pg/ml (S/N = 3). A comparison of various EC methods for the detection of AFP is also listed in Table 1. As revealed, a wider linear range is achieved by the proposed EC method. Although the LOD is not as low as other EC methods, our electrode materials are easily obtained and have simple operation methods.

### 3.5 Anti-interference and stability of the immunosensor

**FIGURE 5:**
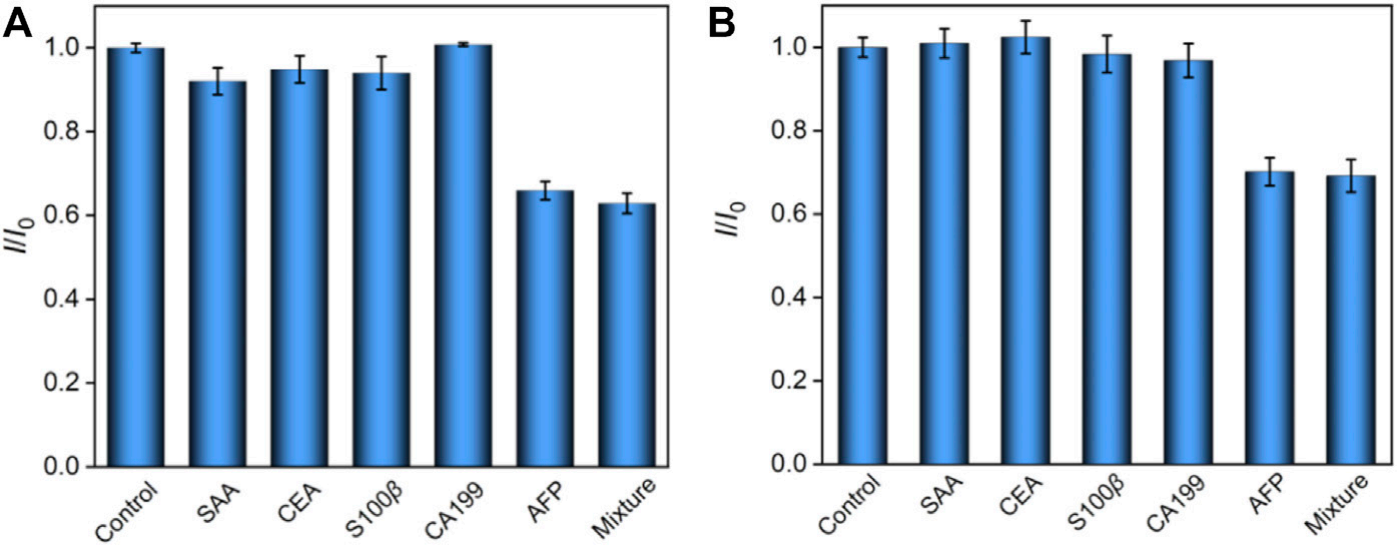
Relative ratio (*I*/*I*_0_) of ECL intensity **(A)** or EC signal **(B)** before (*I*_0_) and after (I) incubation with buffer (control), SAA, CEA, S100*β*, CA199, AFP, or their mixture. The concentrations of all the species are 10 pg/ml and 1 ng/ml for ECL and EC detection, respectively.

A series of potentially existing interfering species were chosen to estimate the selectivity of our ECL/EC dual-mode immunosensor, and the results are shown in Figure 5. After incubation with AFP, serum amyloid A protein (SAA), carcinoembryonic antigen (CEA), S100 calcium-binding protein *β* (S100*β*), carcinoma antigen 199 (CA199), or their mixture, only AFP and a mixture of five species could produce a remarkable decrease at the Ab_AFP_/O-VMSF/ITO sensor for both ECL and EC modes. The aforementioned results show the high selectivity of our dual-mode sensor, which is ascribed to the highly specific recognition between a couple of antibodies and antigens. In addition, our proposed dual-mode immunosensor is stable after 20 days when stored at 4°C.

### 3.6 Analysis of AFP in human serum samples

The practical application of the developed immunosensor is evaluated by determining the concentration of AFP in complex biological samples. Different and known concentrations of AFP are artificially added to the serum of a healthy man for direct analysis. As shown in Table 2, the proposed ECL or EC immunosensor exhibits good recoveries ranging from 91.7% to 104.4% and low RSD values (< 5.1%). We also compared the amounts of a human sample spiked with 1 ng/ml AFP obtained from enzyme-linked immunosorbent assay (ELISA) and our proposed dual-mode immunosensor, which showed good consistency and further proved that both ECL and EC modes have good reliability in real sample analysis. Moreover, owing to the excellent anti-fouling ability of VMSF whose nanopores can filter macromolecules, the as-prepared immunosensor has great potential in complex real sample analysis without tedious pretreatments.

**TABLE 2:** Detection of artificially added AFP in human serum.

Sample	Method	Added^a^	Found^a^	RSD/% (n=3)	Recovery/%
Serum^b^	ECL	0.100	0.104	2.4	104
		10.0	9.17	4.3	91.7
		100	103	3.1	103
	EC	0.100	0.102	3.8	102
		10.0	0.957	4.4	95.7
		100	10.2	5.1	102

^a^The units of AFP, detected by ECL and EC, are pg/mL and ng/mL, respectively.

^b^ 50 times diluted with PBS (0.01 M, pH=7.4).

## 4 Conclusion

In summary, we have reported that the ECL/EC signals of Ru (phen)_3_^2+^ can be significantly improved by the VMSF/ITO electrode and used as the bifunctional probe in solution for the construction of the dual-mode ECL and EC immunosensors. Arising from the negatively charged nanochannel walls of VMSF, a large amount of Ru (phen)_3_^2+^ could be electrostatically preconcentrated onto the electrode surface, which could remarkably increase the sensitivity of Ru (phen)_3_^2+^-based ECL/EC sensors. As a proof of concept, when using a tumor biomarker (AFP) as the analyte, the anti-AFP antibody was covalently immobilized onto the external surface of VMSF/ITO to produce the highly specific sensing interface. On the basis of the fact that the formed immunocomplex could hinder the diffusion of Ru (phen)_3_^2+^ to the underlying electrode surface, the intensities of ECL/EC signals at the fabricated immunosensor were decreased, and the sensitively quantitative determination of AFP was realized with a low LOD and relatively low consumption of Ru (phen)_3_^2+^. Moreover, due to the anti-fouling property of VMSF, the proposed dual-mode immunosensor could be successfully applied to the analysis of human serum, which could extend to a large scope of biomarkers and provide a new strategy for clinical diagnosis.

## Data Availability

The original contributions presented in the study are included in the article/Supplementary Material; further inquiries can be directed to the corresponding author.
