# Supplementary material for: Dual-mode electrochemiluminescence and electrochemical sensor for alpha-fetoprotein detection in human serum based on vertically ordered mesoporous silica films
Source: Front Chem. 2022 Nov 7;10:1023998. doi: 10.3389/fchem.2022.1023998 (PMC9676975; doi:10.3389/fchem.2022.1023998)
Supplement: Supplementary file 1 [file DataSheet1.docx]

**Dual-mode electrochemiluminescence and electrochemical sensor for alpha-fetoprotein detection in human serum based on vertically-ordered mesoporous silica films**

**Haiyun Chen^1,2^, Jie Huang^3^, Rongjing Zhang^1,2*^ and Fei Yan^3*^**

^1^ Shanxi Bethune Hospital, Shanxi Academy of Medical Sciences, Tongji Shanxi Hospital, Third Hospital of Shanxi Medical University, Taiyuan, China;

^2^ Tongji Hospital, Tongji Medical College, Huazhong University of Science and Technology, Wuhan, China

^3^ Department of Chemistry, Key Laboratory of Surface & Interface Science of Polymer Materials of Zhejiang Province, Zhejiang Sci-Tech University, Hangzhou, China.

*** Correspondence:**

Corresponding authors: Sxdyymnk@163.com; feifei19881203@126.com;

Fei Yan: 0000-0002-2822-698X

**Table of Contents**

**S1 CV curves of VMSF/ITO before and after incubation of Ab_AFP_**

**S1 CV curves of VMSF/ITO before and after incubation of Ab_AFP_**

**

**

**Fig. S1** CV curves of VMSF/ITO before (black line) and after (red line) incubation with 10 μg/mL Ab_AFP_ obtained in 0.1 M KCl solution containing 2.5 mM Fe(CN)_6_^3–/4–^.
